# Surveillance of Patulin in Apple, Grapes, Juices and Value-Added Products for Sale in Pakistan

**DOI:** 10.3390/foods9121744

**Published:** 2020-11-26

**Authors:** Shabbir Hussain, Muhammad Rafique Asi, Mazhar Iqbal, Muhammad Akhtar, Muhammad Imran, Agustín Ariño

**Affiliations:** 1 Food Toxicology Laboratory, Nuclear Institute for Agriculture and Biology College (NIAB-C), Pakistan Institute of Engineering and Applied Sciences (PIEAS), Jhang Road, Faisalabad 38000, Pakistan; shabbir.ne@gmail.com; 2 Central Analytical Facility Division (CAFD), Pakistan Institute of Nuclear Science and Technology (PINSTECH), P. O. Nilore, Islamabad 45650, Pakistan; 3 Health Biotechnology Division, National Institute for Biotechnology and Genetic Engineering College (NIBGE-C), Pakistan Institute of Engineering and Applied Sciences (PIEAS), Jhang Road, Faisalabad 38000, Pakistan; hamzamgondal@gmail.com; 4 Soil & Environmental Sciences Division, Nuclear Institute for Agriculture and Biology College (NIAB-C), Jhang Road, Faisalabad 38000, Pakistan; akhtarniab@gmail.com (M.A.); imran1631@gmail.com (M.I.); 5 Instituto Agroalimentario de Aragón—IA2 (Universidad de Zaragoza-CITA), Facultad de Veterinaria, 50013 Zaragoza, Spain

**Keywords:** patulin, apple, grapes, fruit products, occurrence, risk

## Abstract

The prime objective of the current study was to investigate the occurrence of mycotoxin patulin (PAT) in apples, grapes and their value added products. PAT was determined by a validated method based on HPLC with UV detector. A total of 381 samples comprising apple and grape fruits (*n* = 133 each), apple-based products (*n* = 76, juice, puree, jam) and grape juice (*n* = 39) were analyzed. PAT was found in 58.9% samples of apple and apple-based products, with a mean of 49.8 µg/kg (maximum 396 µg/kg), while 27.3% samples contained PAT beyond the maximum regulatory limit of 50 µg/kg. The average levels of PAT contamination in apple-derived products was higher in apple juice concentrate, followed by apple puree, apple juice and apple jam. The incidence of PAT in table grapes was 65.1%, with a mean of 53.9 µg/kg (maximum 505 µg/kg), whereas 23.8% exceeded the maximum level. Among the fruit samples, there were differences in PAT contents due to apple variety (6 types) or grape variety (8 types), as well as for sampling location. Our investigations showed the wide PAT occurrence in fruits and derived value-added products affecting consumer product safety, so that the population is chronically exposed to this toxin.

## 1. Introduction

Pakistan is located in South Asia with an extremely diverse climate that varies from tropical to temperate and even cold in the northern part. The diversity of the landscape and climate in Pakistan allows a wide variety of fruit trees that produce fruits with good flavor and taste, which are available throughout the year. Apple fruits are cultivated and harvested in the northern geographic area whereas grapes are mainly grown in the central part of the country. The different fruit varieties are cultivated in an area of around 800,000 hectares (11% Ha under apples and 2% Ha for grapes) with a total production of 7.05 million tons (8% apples and 1% grapes) [1]. During the 2017–2018 crop season, only 10% of the entire fruit production was exported. Fruits are generally high in fiber, vitamin C, beta-carotene and antioxidants, and have a significant role in the human diet, as their intake is associated with a reduced risk of cardiac disease, all-cause mortality and cancer [2]. Apples and grapes are eaten fresh and are preserved as jams, as well as processed as fruit juices or used as ingredients in many manufactured foods.

Many fruits are perishable and susceptible to fungal attack that cause maceration and decay that reduces quality, and can lead to the synthesis of mycotoxins like patulin (PAT). This mycotoxin is frequently linked with fruits, their juices and value added products comprising foods for children, and it is a common contaminant of apple and apple-based products [3]. Patulin mycotoxin is primarily produced by toxigenic strains of the fungi *Penicillium expansum*, *Aspergillus clavatus* and *Byssochlamys nivea*, though the blue-mold disease, triggered by *P. expansum*, is the utmost postharvest disease of fruits in storage [4]. This fungus can infect fruits before or during harvest via injuries, with the subsequent patulin production during post-harvest [5]. Conventional techniques for PAT testing have used multi-step liquid–liquid extraction (LLE), solid-phase extraction (SPE) or molecularly imprinted polymers (MIP), followed by determination by liquid chromatography with ultraviolet, diode array or mass spectrometry detection [6].

There are numerous aspects that affect patulin incidence and its contamination in fruits, such as climate conditions, type and cultivar of fruits, year of production, geographical location, pre- and post-harvest managements, storage environments and surface damage on the fruits [7]. Snini et al. [8] showed that PAT is a cultivar-dependent factor supporting the colonization of apple fruits by *P. expansum*. Among thirteen different apple varieties, the majority showed significant differences in the progress of fungal decay and the total quantities of PAT. The cultivar influence for patulin in grapes has not been previously identified although it has been extensively studied in wine grapes for other mycotoxins such as ochratoxin A [9].

Patulin has been stated to cause gastrointestinal disorders with ulceration and bleeding, as well as genotoxicity, neurotoxicity, hepatotoxicity and immunotoxicity [10]. Because of the toxicological implications of PAT, several regulatory institutions worldwide have established maximum levels of patulin, mainly in the apple-based products, ranging from 25 to 50 µg/kg. The Codex Alimentarius [11] set the maximum permissible PAT levels in fruits and their juices at 50 µg/kg. According to the Commission Regulation (EC) No. 1881/2006, the European Union (EU) fixed limits of patulin in fruit juices (50 µg/kg), solid apple products (25 µg/kg) and foods for infants and young children (10 µg/kg) [12].

In view of the above facts, the focus of the current research was to assess the incidence of patulin in apple and grape fruits, along with their value added products, collected during two years in Pakistan, and compare the levels with the regulatory maximum limits. In addition, the outcomes of the present survey will also allow decisions on control strategies to be taken derived from objective data.

## 2. Materials and Methods

### 2.1. Sampling

Random samples of fresh fruits, juices and derived products (purees and jams) for sale were procured from city markets, supermarkets and general stores from different areas of Pakistan during the years 2018 and 2019. A total of 133 samples of fresh apple fruits designated for direct consumption were taken, out of which 70 from different varieties (Amri 9, Kashmiri 11, Kala Kulu 15, Golden delicious 14, Red delicious 13, and Gaja 8) and 63 samples from different cities (Rawalpindi 10, Faisalabad 15, Lahore 8, Gilgit 7, Swat 8, Muzafarabad 9 and Murree 6). For the study of the different apple varieties, the samples were collected from orchard owners and from fruit and vegetable wholesale market in Faisalabad. In Pakistan, the apple varieties are mostly cultivated in Baluchistan and Khyber Pakhtunkhwa provinces of Pakistan, as well as in Azad Jammu and Kashmir. The fruits from these areas are transported throughout the country, especially to major cities. For the study of the different sampling locations, the apple samples were directly purchased from vendors and fruit shops located in the vicinity of the cities, but the variety was not specifically recorded because the sellers did not have such information. Additionally, 76 samples of apple-derived products (juice 35, concentrate 10, puree 21, jam 10) belonging to commercial brands of nation-wide distribution were taken. Similarly, a total of 133 samples of fresh table grapes were sampled, out of which there were 79 from different varieties (White Thomson 6, King ruby 9, Narc black 12, Perlette 8, Sundar Khani 14, Vitro black 10, Cardinal 9, Flame 11) and 54 samples from different cities (Faisalabad 16, Lahore 10, Rawalpindi 10, Attock 7, Dunyapur 5 and Chakwal 6). For the study of the different grapes varieties, the samples were collected from orchard owners and from a fruit and vegetable wholesale market in Faisalabad. In Pakistan, the table grape varieties are mainly cultivated in central Punjab, Baluchistan and Khyber Pakhtunkhwa provinces of Pakistan, and transported to other regions of the country. For the study of the different sampling locations, the grape samples were procured from vendors and fruit shops located in the vicinity of the cities, but the sellers did not provide varietal information. In addition, 39 samples of grape juice from different brands were taken. Fresh fruits (1 kg), fruit juices (1 L) and derived products (500 g package) were stockpiled at 4 °C in their original packages in Food Toxicology Laboratory at NIAB Faisalabad until analysis. All the stored samples were opened and homogenized thoroughly before analysis.

### 2.2. Chemicals and Reagents

HPLC grade glacial acetic acid, acetonitrile, and ethyl acetate, and analytical-grade sodium chloride, sodium sulfate anhydrous and sodium carbonate were purchased from Merck (Darmstadt, Germany). Patulin standard, 5-hydroxymethyl furfural (5-HMF) and pectinase enzyme solution were supplied by Sigma-Aldrich (Saint-Louis, MO, USA). Stock reference standard solution of PAT 1.0 mg/mL was prepared in HPLC-grade acetonitrile and stockpiled at −4 °C. Required volumes of working standard solutions (5, 10, 30, 50, 70, and 100 µg/L) were prepared from a stock solution in 0.1% acetic acid. Multifunctional cleanup columns PriboFast^®^, MFC-228 were obtained from Pribolab (Singapore).

### 2.3. Samples Preparation, Extraction and Cleanup

The procured samples of fruits were selected without debris, washed adequately with tape and distilled water, dried in the shade and cut into small slices using a sharp knife. Around 500 g sample was thoroughly homogenized by a high-speed mixer blender (Mix 2000, Braun Blender, Marktheidenfel, Germany). All samples of fruit products (juice, jam and puree) were directly taken from original stored packages. The analytical technique for PAT assay was established based on AOAC, method 2000.02 [13]. For fresh fruits and clear juices, no preparation was required. For cloudy juices, such as puree and jam, a sample portion (25 mL or 25 g) was pretreated with 150 µL pectinase enzyme and incubated for 2 h at 40 °C to clarify the sample prior to extraction. Homogenized fruit and product samples (25 mL or 25 g), were extracted two times using 50 mL ethyl acetate accompanied by 2 g sodium chloride in the 250 mL Erlenmeyer flask. The contents of flask were shaken using a horizontal shaker (Gunther, and Co, Bremen, Germany) at a high speed for about one hour. The organic upper layers (combined) were shaken with 10 mL of sodium carbonate (1.5%, Na_2_CO_3_ solution). The lower aqueous layer was drained and the top layer was quickly dehydrated using 15 g of anhydrous powder sodium sulfate (Na_2_SO_4_), and then filtered by using filter paper (Whatman No. 1). The filtrate was subjected to clean-up through MFC 228 multifunctional column, and purified extract 4 mL was collected in liquid scintillation vial. The extracts of samples were evaporated under stream of nitrogen to dryness. The dried residue was instantly dissolved in 1 mL of 0.1% acetic acid, and filtered through a 0.22 µm syringe filter (Millipore, Darmstadt, Germany) to get a clear solution. The extracted samples were then analyzed for the PAT assay by reverse phase HPLC system equipped with ultraviolet-Vis detector (SPD 10AS, Shimadzu, Japan) using an isocratic mode of operation. The total run time was set at 10 min and 20 µL volume of sample was injected for PAT analysis.

### 2.4. Apparatus and Conditions of HPLC for Analysis

The apparatus used for the present study was HPLC (LC-10A Shimadzu, Kyoto, Japan) equipped with UV-Visible detector (SPD-10AS) at a wave length of 276 nm. Separations were performed using Discovery HS C-18 silica-based column (250 × 4.6 mm, particle size 5 µm; Supelco, Bellefonte, PA, USA), temperature sustained at 30 °C with 1.5 mL/min flow rate in isocratic mode. The mobile phase used was a mixture of acetonitrile and water (10:90, *v*/*v*). A Rheodyne injector (20 µL loop) was used for injection of standards and sample extracts, and Software CLASS LC-10 (version 1.63, Shimadzu, Kyoto, Japan) was used for acquisition of chromatograms. Patulin was confirmed by its retention time (≈6.558 min) according to a reference standard, and quantified by measuring the peak area according to a standard curve. For validation method, a simple test was executed to confirm the separation of PAT peak and 5-HMF peak that is its principal interference.

### 2.5. Statistical Analysis

Statistical analysis was executed for the compiled data. The Excel software was used to calculate descriptive statistics of patulin in fruits, their juices and their derived products and data were presented as a mean along with standard deviation. The samples with levels of PAT greater than the limit of detection (LOD) were considered as positive. The mean was calculated considering zero value, for the samples with PAT levels less than LOD. The significance differences in patulin concentrations between the groups were investigated using a one-way ANOVA (*p* < 0.05) test, by SPSS (IBM, SPSS Statistics, 19, Armonk, NY, USA, 2010).

## 3. Results and Discussion

### 3.1. Method Validation

The HPLC-based analytical method was evaluated in terms of linearity, recovery, precision and sensitivity. Linearity was tested by injection of patulin standards at concentrations in the range of 5 to 100 µg/L, obtaining a correlation coefficient of 0.9921. Recovery experiments were operated on spiked samples at different PAT levels (10, 20, 50, 70 and 100 µg/kg). The mean recoveries of PAT in spiked apples (*n* = 3) were 96.5, 94.3, 95.1, 100.5 and 101.1%, respectively (mean value 97.5%), while recoveries for spiked grapes attained 92.8, 94.0, 93.2, 100.0 and 96.0%, respectively (mean value 95.2%). Intra-day (*n* = 3) and inter-day (3 different days) variation values ranged between 4.3 and 7.2% (RSD_r_) and from 4.7 to 7.4% (RSD_R_), respectively, indicating good precision. The detection limit (LOD) and the limit of quantification (LOQ) values were calculated according to s/*n* = 3 and s/*n* = 10, respectively. The LODs and the LOQs of PAT were 5 μg/kg and 15 μg/kg, respectively. The performance criteria for patulin set out in Commission Regulation (EC) No. 401/2006 [14] establishes three set of values, (1) Level < 20 µg/kg: recovery 50 to 120%, RSDr ≤ 30, RSDR ≤ 40, (2) Level 20–50 µg/kg: recovery 70 to 105%, RSDr ≤ 20, RSDR ≤ 30, and (3) Level > 50 µg/kg: recovery 75 to 105%, RSDr ≤ 15, RSDR ≤ 25. Conclusively, the used analytical method fulfilled the performance criteria of Commission Regulation (EC) No. 401/2006 [14] for the accurate determination of patulin levels in foodstuffs.

### 3.2. Occurrence of Patulin in Apple Fruits and Their Products

Incidence and contamination level of PAT in apple fruits and apple-based products are given in Table 1. The results revealed that 123 out of 209 samples (58.9%) showed positive for PAT. The positivity in fresh apple fruits was 56.4%, while apple-derived products attained 63.2%. From the six apple fruit varieties analyzed, the prevalence varied from 26.7% (Kala Kulu) to 100% (Gaja), while average PAT concentrations were between 32.3 µg/kg (Kala Kulu) and 107.3 µg/kg (Gaja). However, the differences between apple varieties were not significant (ANOVA *p* = 0.4443). In turn, there were significant differences in PAT levels depending on sampling location (ANOVA *p* = 0.0162), ascribed to high levels in samples from Murree. The apples coming from this mountain city in the northeast of the country, showed a PAT incidence of 100% with average levels of 183.8 µg/kg, while those from other locations showed incidence from 25 to 70% and lower levels between 9.4 to 59.2 µg/kg.

PAT was also examined in samples of apple-based products, the incidence of which ranged from 30% in apple jam to 80% in apple juice concentrate. Similarly, the levels were much higher in apple juice concentrate (107.5 µg/kg) than in other products that ranged from 1.7 to 29.4 µg/kg. The maximum level of PAT was found in an apple fruit from Murree (396 µg/kg), followed by a sample of apple juice concentrate that amounted to 328 µg/kg. Overall, 57 samples of apple fruits and derived products (27.3%) surpassed the maximum regulatory limit of 50 µg/kg.

Patulin is a toxic substance produced by molds that may grow on apples. The patulin quantity detected in apple products is generally taken as a measured value for the quality of the apples used for the food production. In several studies worldwide, patulin has been found to occur at high levels in apple fruits and derived products offered for sale (Table 2). In a previous survey carried out in Pakistan, high levels of PAT in apple fruits and apple juices were reported by Iqbal et al. [15] PAT was found in apple fruits (36 samples, 66.7% positive) at 259 µg/kg average level, while mean level in apple juice (29 samples, 51.7% positive) was 26 µg/kg, which is in accordance with the present findings. In an extensive survey carried out in Argentina, PAT showed positive in 40.3% of 4634 samples of apple fruits, with an average level of 26 µg/kg and a maximum value of 19,622 µg/kg [16]. Vaclavikova et al. [17] carried out a survey of patulin in fresh fruits and derived products for sale in the Czech Republic. In fresh apples and apple pulp, PAT levels varied from 1.3 to 415.2 µg/kg, while in apple juice they were from 3.8 and 28.4 µg/kg. A study conducted by Al-Hazmi in Saudi Arabia [18] reported patulin in apple juice in the range LOD–152.5 µg/kg. In all mentioned studies, researchers have found the toxin in concentrations beyond the permitted levels. All these findings are well supportive to our results of concentration of PAT in apple fruit and apple-based products.

The high incidence of patulin in apple samples is in line with the survey for postharvest diseases of apples conducted in Punjab (Pakistan), which revealed an occurrence of blue mold caused by *Penicillium expansum*, which was found with the highest prevalence (56%) [19]. Patulin contamination is most common on spoiled fruits, often intended for juices and preserves. Its occurrence has been a major issue in the processing of apple juices, as due to its thermal stability, patulin is not destroyed during pasteurization [6]. However, physical adsorption of PAT by the yeast *Saccharomyces cerevisiae* during the process of fermentation has been reported [20].

Patulin is a mycotoxin produced by fungi belonging to several genera, including *Penicillium*, *Aspergillus* and *Byssochlamys* species. Although patulin can occur in many moldy fruits, grains and other foods, the major sources of patulin contamination are apples and apple products. The presence of molds and mycotoxins play an important role in the depreciation of quality and safety of apples and their derived products. Toxigenic fungi infect fruits either prior to or after harvesting and during transportation, handling, storage and marketing conditions that make fruits prone to fungal deterioration and decay. Filamentous fungi can enter fruits through damaged surfaces, like splits, wounds, cuts and punctures. *Penicillium expansum*, that produces PAT, has been reported as a major causative agent of post-harvest decay in fruits in Pakistan [21].

**Table 2:** A summary of studies on PAT occurrence in apple fruits and derived products.

Commodity	Country	Analytical Method	Incidence (%)	Concentration Range (µg/kg)	Reference
Apple juice for infants	Spain	MEKC ^1^	70	LOD–29.6	Murillo et al. [22]
Apple juice concentrate	Spain	HPLC-UV ^2^	42.4	LOD–74.4	Marín et al. [23]
Apple based food	Serbia	HPLC-UV	43	3.2–30.2	Torović et al. [24]
Apple juice	Sweden	HPLC-UV	12.8	LOD–50	Thuvander et al. [25]
Apple juice	Italy	HPLC-UV	37.5	5.8–56.4	Ritieni et al. [26]
Apple puree	Italy	HPLC-UV	50	15.9–16.7	Ritieni et al. [26]
Apple juice	Portugal	HPLC-DAD ^3^	41	1.2–42	Barreira et al. [27]
Apple juice	Belgium	HPLC-UV	12	10.2–43.1	Baert et al. [28]
Apple juice	Greece	HPLC-DAD	100	0.9–36.8	Moukas et al. [29]
Apple juice	Turkey	HPLC-UV	60	19.1–732.8	Yurdun et al. [30]
Apple juice	Tunisia	HPLC-UV	64.3	4–122.4	Zouaoui et al. [31]
Apple juice	Tunisia	HPLC-UV	37	0–167	Zaied et al. [32]
Apple juice	S. Africa	HPLC-UV	23.5	5–45	Leggot et al. [33]
Apple juice	S. Africa	HPLC-UV	33.3	0–1650	Shephard et al. [34]
Apple juice	USA	HPLC-DAD	18.7	LOD–467.4	Harris et al. [35]
Apple juice	Brazil	HPLC-DAD	3	3–7	Iha et al. [36]
Apple fruit	Argentina	HPLC-DAD	40.3	LOD–19622	Oteiza et al. [16]
Apple juice	Malaysia	HPLC-UV	7.7	LOD–26.9	Lee et al. [37]
Apple juice	China	LC-MS ^4^	42.9	LOD–1234.3	Li et al. [38]
Apple juice	Japan	LC-MS	19.7	1.4–45.6	Ito et al. [39]
Apple juice	S. Korea	HPLC-DAD	12.5	LOD–8.9	Cho et al. [40]
Apple juice	Iran	TLC ^5^	31	15–285.5	Cheraghali et al. [41]
Apple fruit	Pakistan	HPLC-UV	56.4	LOD–396	Present study
Apple juice	Pakistan	HPLC-UV	62.9	LOD–18	Present study
Apple juice concentrate	Pakistan	HPLC-UV	80	LOD–328	Present study
Apple puree	Pakistan	HPLC-UV	71.4	LOD–99	Present study
Apple jam	Pakistan	HPLC-UV	30	LOD–6	Present study

^1^ Micellar electrokinetic chromatography; ^2^ Liquid chromatography with ultraviolet detection; ^3^ Liquid chromatography with diode array detection; ^4^ Liquid chromatography with mass spectrometry detection; ^5^ Thin-layer chromatography.

The occurrence of PAT in analyzed apple varieties showed some differences that may be due to several factors, such as apple texture firmness. Previous research on PAT accumulation in numerous apple cultivars at different times during storage indicated a dependence on cultivar [42]. In Pakistan, an in vitro study conducted by Sattar et al. [43], reported that Golden Delicious (locally Shin Kulu) showed significantly higher growth of *P. expansum* as compared to Red Delicious (locally Tor Kulu). The authors attributed the different susceptibility to blue mold in that Golden Delicious apples have a thin cuticle and the least amount of acids and are prone to more injuries as compared to other varieties.

The factors and inducers modulating patulin synthesis in apples are still not clear, although several environmental factors and intrinsic characteristics of the fruits have been suggested to contribute to patulin accumulation [5]. Differences in susceptibility to fungal pathogens among apple cultivars might be related to the apple fruit intrinsic factors such as the content of sugars, ethylene, organic acids and phenolic compounds as well as the pH [44]. Among the thousands of apple cultivars, some of them show a fast response toward wounding and decaying, while other susceptible ones fail to combat pathogen attack, resulting in a large accumulation of patulin within the fruit flesh [7].

Patulin occurrence in apples is probably more affected by weather conditions before harvest and apple storage temperature than by cultivar. Higher storage temperatures will increase the probability for patulin occurrence in apple cultivars whose fruits have thin and fragile skin, but not in firm apples with thicker skin and higher flesh firmness [45]. Therefore, flesh firmness is an important sensory trait of all apple cultivars, particularly domesticated ones, that is associated with the fruit resistance to blue mold decay and patulin production [7]. In addition, the presence of a bitter pit, a physiological disorder of apples related to nitrogen fertilization and intensive pruning of trees, can weaken the resistance of the fruit to pathogens favoring the growth of patulin-producing fungi [45]. Likewise, phenolic compounds can have an important role as defense mechanisms against pathogens and patulin in apple cultivars [46]. All these parameters are contributing factors for the fungal deterioration and decay of apple fruits and mycotoxin production. Therefore, many factors affect the levels of contamination by patulin in apples, and it is difficult to determine which of them have been decisive in each case. However, climatic conditions before harvest (i.e., rainfall two weeks before harvest), during transportation, storage and handling, as well as retailer training are key factors that cannot be ignored. All these elements are responsible for the variation in PAT levels in the analyzed samples.

Patulin is extremely soluble in water, and highly stable at an acidic pH, so it can migrate beyond lesion of fungal attack in stored fruits during post-harvest. Therefore, cleaning of fruits only by washing and exclusion of decayed parts cannot guarantee PAT decontamination. For instance, Olsen et al. [47] reported PAT contamination in apple jam due to surface growth of *P. expansum* and observed PAT penetration up to 4 cm layer of the jam. The proper handling and storage of fruits is the basis for the prevention of PAT [48]. In this sense, the storage temperature is a key factor to explain patulin accumulation in apples, as the higher the storage temperature, the greater the mycotoxin accumulation [5,43]. In order to increase the quality of apples in Pakistan, there is a need to establish treatment plants and cold-storage facilities for fruit preservation during off-season.

Our study indicated that patulin is frequently detected in apple and apple products throughout Pakistan and the degree of contamination is affected by multiple factors, so further studies with different cultivars in different pre- and postharvest conditions should be carried out, in order to better understand the risk for its occurrence in apple products.

### 3.3. Occurrence of Patulin in Grapes Fruits and Their Products

Incidence and contamination level of PAT in grape fruits and juices are shown in Table 3. The results revealed that 112 out of 172 samples (65.1%) were positive for PAT. The positivity in fresh table grapes was 59.4%, while in grape juices it reached 84.6%. From the eight grape varieties analyzed, the prevalence varied from 50% (White Thomson) to 66.7% (King Ruby), while the average PAT concentrations were between 33.8 µg/kg (White Thompson) and 90.9 µg/kg (Sundar Khani). Nevertheless, the differences between grapes varieties were not significant (ANOVA *p* = 0.9458). As regards of sampling location, the incidence varied between 50% (Lahore) and 70% (Rawalpindi), while average concentrations ranged from 22.5 µg/kg (Attock) to 101.1 µg/kg (Rawalpindi), but differences were not significant (ANOVA *p* = 0.7258).

PAT was also found in 33 out of 39 grape juice samples (84.6%) from the market, at a mean concentration of 16.3 µg/kg. The highest level of PAT was found in a sample of grapes from Faisalabad (505 µg/kg), followed by a sample of Sundar Khani grapes that amounted to 490 µg/kg. Overall, our results highlighted that 41 samples of grapes fruits (23.8%) surpassed the maximum regulatory limit of 50 µg/kg, while all grapes juice samples were compliant.

Patulin has been mainly associated with apples, and apple-based products. However, the toxin may possibly contaminate the other fruits, such as grapes (Table 4). For instance, PAT was shown to be positive in 5 out of 50 samples of grapes fruits from Argentina, with a mean level of 283 µg/kg and a maximum value of 13,808 µg/kg [16]. In a previous survey carried out in Pakistan [15], high levels of patulin in grapes fruits were also determined. They found PAT in 22 out of 31 samples (71% positive) but average level of 504 µg/kg was much higher than our findings.

The presence of patulin-producing strains of *Penicillium* has been previously reported in grapes. Sanzani et al. [49] in Italy isolated *Penicillium* DNA from samples of grape must and wine contaminated with patulin (27 to 1911 µg/kg). Ostry et al. [50] verified the occurrence of PAT in grape must in Czech Republic, where contamination levels varied between 119 and 644 µg/kg.

In our study, mean PAT concentration was higher in grapes with seeds (Sundar Khani and Vitro Black) than in seedless varieties such as White Thomson. It has been reported that the absence of seeds in some grape cultivars likely results in a low content of antifungal metabolites such as tannin in fruits, making these grapes more susceptible to infection. During in vitro inoculation with *Aspergillus carbonarius*, progression of the rotted area of the fruit and OTA accumulation was the fastest for Thompson seedless grapes, as compared to other varieties [9]. For most grape cultivars, mold contamination was greater in rotten berries than in intact berries [51]. Therefore, keeping the berries intact is likely to be essential for the storage of table grapes.

Fungal colonization and mycotoxin production on grapes are influenced by several factors, including environmental conditions, location of the vineyard, and the characteristics of the grapes. Differences in susceptibility to fungal pathogens among grape cultivars might be related to the physical and chemical properties of grape berries. It has been found that the thickness of the berry skin, pH, the content of reducing sugars, soluble solids and organic acids contribute to the resistance of grape berries to fungal invasion and development [52].

The fungal attack also depends on the climate circumstances, improper conditions throughout the storage, transportation and sale, resulting in decayed grapes. The grapes are highly susceptible to *Penicillium* species that contribute to PAT production through deterioration and decay, especially during storage [53]. It is likely that the fruit vendors did not care about the storage conditions due to a lack of knowledge and proper training required for enhancing the shelf life of fruits and their products.

In Pakistan, consumption of apple, grapes and value-added products contaminated with PAT is a matter of worry for the associated health problems. In addition, patulin is a priority pollutant in products of fruit and vegetables used for processing in global trade. Then, the presence of toxins could be a source of economic losses and failure to achieve the United Nations Sustainable Development Goals (SDGs). The findings of the present study could help to increase awareness of the problem, and to better the understanding of PAT contaminations in apple, grapes and their products among farmers, traders, end-users and regulatory authorities of Pakistan.

The occurrence of filamentous fungi in grapes may result in mycotoxins, including patulin, ochratoxin A, aflatoxins, fumonisin B2, citrinin, alternariol and tenuazonic acid, among others [54]. However, the focus of the present study was to assess the levels of PAT in fruits that are currently exported to other countries as well as consumed at gross level in Pakistan.

**Table 4:** A summary of studies on patulin occurrence in grapes fruits and derived products.

Commodity	Country	Analytical Method	Incidence (%)	Conc. Range (µg/kg)	Reference
Grapes fruit	Argentina	HPLC-UV	10	0–13,808	Oteiza et al. [16]
Grapes juice	Austria	HPLC-UV	45.3	LOD–41	SCOOP Task [55]
Grapes must	Austria	HPLC-UV	52.4	LOD–750	SCOOP Task [55]
Grapes juice	Belgium	HPLC-UV	20	LOD–36	SCOOP Task [55]
Grapes juice	Germany	HPLC-UV	3.1	LOD–31.5	SCOOP Task [55]
Grapes must	Germany	HPLC-UV	54	3.5–80	Majerus at al. [56]
Grapes juice	Germany	GC-MS	100	4.9–5.2	Rychlik et al. [57]
Grapes fruit	Pakistan	HPLC-UV	59.4	LOD–505	Present study
Grapes juice	Pakistan	HPLC-UV	84.6	LOD–39	Present study

## 4. Conclusions

The validated analytical methodology for the determination of patulin based on HPLC with UV detection exhibited good sensibility, accuracy and precision and might be used for routine quality control and quality assurance of fruits and their products. The recognition of PAT as a hazard has risen in Pakistan over the last decade. This study provides detailed information on the risk associated with PAT in apples, grapes and their products, which poses a potential health threat to consumers due to its toxic effects. Not in vain, a significant percent of analyzed apple (27.3%) and grape (23.8%) samples exceeded the maximum regulatory level set at 50 µg/kg. The findings of the current study would be useful for producers, processors and regulatory authorities to introduce policies for the prevention and control of PAT in fruits before launching to the market or exporting. We recommend regular monitoring of the incidence of PAT to maintain food safety and minimize impact on trade. Surveys on the incidence and levels of mycotoxins are of prime importance because they are reliable approaches to the current incidence of these chemical contaminants in foodstuffs. They are relevant for food safety and toxicology as the results can be compared with previous studies and make it possible to assess the effect of various factors on the occurrence of mycotoxins.

## Figures and Tables

**Table 1:** Occurrence of patulin (µg/kg) in apple fruits and their derived products.

Sample Type	Variety/Location	*n* Total (Positive)	Incidence %	Mean ± SD	Max.	*n* (%) > 50 µg/kg
Apple fruit	Amri	9 (5)	55.6	66.9 ± 94.7	221	3 (33.3)
Apple fruit	Kashmiri	11 (7)	63.6	100.6 ± 111.3	264	5 (45.5)
Apple fruit	Kala Kulu	15 (4)	26.7	32.3 ± 82.2	308	3 (20)
Apple fruit	Golden delicious	14 (9)	64.3	68.1 ± 74.1	189	6 (42.9)
Apple fruit	Red delicious	13 (6)	46.2	67.6 ± 103.4	276	5 (38.5)
Apple fruit	Gaja	8 (8)	100.0	107.3 ± 106.1	299	4 (50)
Apple fruit	Rawalpindi City	10 (7)	70.0	55.3 ± 68.4	182	4 (40)
Apple fruit	Faisalabad City	15 (10)	66.7	52.2 ± 75.7	283	6 (40)
Apple fruit	Lahore City	8 (4)	50.0	57.9 ± 102.3	277	2 (25)
Apple fruit	Gilgit City	7 (3)	42.9	16.9 ± 35.6	97	1 (14.3)
Apple fruit	Swat City	8 (2)	25.0	9.4 ± 24	68	1 (12.5)
Apple fruit	Muzafarabad City	9 (4)	44.4	59.2 ± 110.7	311	2 (22.2)
Apple fruit	Murree City	6 (6)	100.0	183.8 ± 144.2	396	5 (83.3)
Apple juice	General stores	35 (22)	62.9	5.6 ± 5.1	18	0 (0)
Apple juice concentrate	Supermarket	10 (8)	80.0	107.5 ± 115.5	328	5 (50)
Apple puree	City market	21 (15)	71.4	29.4 ± 32.3	99	5 (23.8)
Apple jam	General stores	10 (3)	30.0	1.7 ± 2.7	6	0 (0)
Total	---	209 (123)	58.9	49.8 ± 83.6	396	57 (27.3)

**Table 3:** Occurrence of patulin (µg/kg) in grapes fruits, and their derived products.

Sample Type	Variety/Location	*n* Total (Positive)	Incidence %	Mean ± SD	Max.	*n* (%) > 50 µg/kg
Grapes fruit	White Thompson ^1^	6 (3)	50	33.8 ± 50.3	119	2 (33.3)
Grapes fruit	King Ruby ^1^	9 (6)	66.7	52.7 ± 60.6	152	4 (44.4)
Grapes fruit	Narc Black ^1^	12 (7)	58.3	60.4 ± 93.2	274	4 (33.3)
Grapes fruit	Perlette ^1^	8 (5)	62.5	53.4 ± 102.3	298	2 (25)
Grapes fruit	Sundar Khani	14 (8)	57.1	90.9 ± 140.9	490	6 (42.9)
Grapes fruit	Vitro Black	10 (6)	60	63.6 ± 112.9	356	3 (30)
Grapes fruit	Cardinal ^1^	9 (5)	55.6	62.9 ± 114.2	310	2(22.2)
Grapes fruit	Flame ^1^	11 (6)	54.5	40.5 ± 68.8	183	2 (18.2)
Grapes fruit	Faisalabad City	16 (10)	62.5	99.4 ± 169.2	505	5 (31.3)
Grapes fruit	Lahore City	10 (5)	50	57.7 ± 107.1	306	2 (20)
Grapes fruit	Rawalpindi City	10 (7)	70	101.1 ± 139.7	416	4 (40)
Grapes fruit	Attock City	7 (4)	57.1	22.5 ± 38.7	107	1 (14.3)
Grapes fruit	Dunyapur City	5 (3)	60	55 ± 89.2	210	2 (40)
Grapes fruit	Chakwal City	6 (4)	66.7	44.6 ± 70.7	180	2 (33.3)
Grapes juice	Supermarket	39 (33)	84.6	16.3 ± 11.9	39	0
Total	---	172 (112)	65.1	53.9 ± 98.5	505	41 (23.8)

^1^ seedless grapes varieties.
